# Determinants of Coinfection in the Mycoviruses

**DOI:** 10.3389/fcimb.2019.00169

**Published:** 2019-05-24

**Authors:** Vaskar Thapa, Marilyn J. Roossinck

**Affiliations:** Department of Plant Pathology and Environmental Microbiology, Center for Infectious Disease Dynamics, Pennsylvania State University, University Park, PA, United States

**Keywords:** mixed infections, fungal virus, non-self-recognition, mycovirus transmission, virus symbiosis

## Introduction

Coinfections of mycoviruses are generally common. The coinfecting mycoviruses are not necessarily the result of horizontal virus transmission among homologous fungal hosts compatible for anastomosis, but involve mycoviruses from phylogenetically diverse sources (Herrero and Zabalgogeazcoa, 2011; Osaki et al., 2016; Ran et al., 2016; Hao et al., 2018). An experimental study with different combinations of transmission scenarios among four partitiviruses showed a significant positive influence of one virus (Heterobasidion partitivirus13-an1) on its distantly related coinfecting partner Heterobasidion partitivirus 15-pa1, but no support of coinfection between two related viruses (Heterobasidion partitiviruses 11-au1 and 11-pa1) (Kashif et al., 2019). A more stable coinfection between distantly related species than conspecific strains was also reported among mycoviruses infecting the fungus, *Heterobasidion parviporum* (Vainio et al., 2015). Studies covering large geographical areas indicate that the mycoviruses in coinfections belong to the local fungal community (Ran et al., 2016; Arjona-Lopez et al., 2018). Arjona-Lopez et al. (2018) found a non-overlapping set of coinfecting mycoviruses in isolates of *Rosellinia necatrix* from Japan and the Mediterranean that match with mycoviruses from the respective local fungal pools. In general, transmission of mycoviruses occurs through anastomosis of vegetatively compatible strains of the same species, but phylogenetic evidence implies occasional transmission across vegetatively incompatible strains. The transmissions across vegetatively incompatible fungal hosts are poorly studied except in a few cases (Liu et al., 2003, 2016; Yaegashi et al., 2013a). In addition to transmission across heterologous fungi, interactions among mycoviruses play direct roles in coinfection. The interactions among coinfecting mycoviruses are diverse, ranging from synergistic to neutral to antagonistic. In some cases, mycovirus coinfection induces genome rearrangement of one of the coinfecting partners, likely through recombination (Sun and Suzuki, 2008). A more comprehensive review on the interactions among coinfecting mycoviruses is found in Hillman et al. (2018).

The diversity of coinfecting mycoviruses and the diverse nature of interactions may imply that coinfections occur freely without any constraints. However, in examples from *in vitro* experiments the frequency of coinfection varies with different fungal systems and in many cases is lower than what would be expected from random incidences. For example, in 43 isolates of the ascomycete *Tolypocladium cylindrosporum*, coinfections of mycoviruses were reported in only in about 5% of the population (Herrero and Zabalgogeazcoa, 2011). In contrast, in almost 200 isolates of *Ustilaginoidea virens* examined multiple dsRNA elements were found in large samples, and the coinfection frequency of two common mycoviruses: Ustilaginoidea virens RNA virus 1 (*Totiviridae*) and Ustilaginoidea virens RNA virus 4 (unclassified) was close to 30% (Jiang et al., 2015). In our own study of about 200 North American isolates of *Pseudogymnoascus destructans* a fungus causing a deadly disease in bats, the coinfection incidence of a partitivirus (Thapa et al., 2016) and an unclassified mycovirus is close to 25%, but only in a geographically restricted area (unpublished). Independent segregation of coinfecting mycoviruses in the population has been described only in a few cases where the interaction is likely neutral, while in other cases one of the partners influences the presence of other. In the coinfection of two unrelated RNA viruses, Yado-nushi virus 1 (YnV1) and Yado-kari virus 1 (YkV1) in *Rosellinia necatrix*, YkV1 is dependent on YnV1. YkV1 does not segregate independently in the population (Zhang et al., 2016). Thus, the coinfection scenarios are likely influenced by the interactions between coinfecting viruses or at the level of mycovirus transmission, particularly during the transmission across vegetatively incompatible fungi or from other factors. Such constraints led us to investigate the determinants of mycovirus coinfections. Here, we discuss some of the determinants for mycovirus coinfection based on the literature available and provide our opinions on coinfection biology.

## Mycovirus Associated Suppression of Fungal Non-Self-Recognition

This is an example of a coinfection system where one of the mycoviruses suppresses its fungal host's non-self-recognition, which facilitates heterologous transmission of mycoviruses. Non-self-recognition or allorecognition is a ubiquitous phenomenon in fungi that enables them to distinguish one from another (Glass and Dementhon, 2006). In fungi, non-self-recognition between two isolates of different mycelial compatibility results in compartmentalization followed by programmed cell death to interrupt fusion between hypha, termed as heterokaryon incompatibility (Glass and Kaneko, 2003). Sclerotinia sclerotiorum mycoreovirus 4 (SsMYRV4), which is associated with hypovirulence in *Sclerotinia sclerotiorum*, was found to suppress non-self-recognition of the fungus and facilitate coinfection through horizontal transmission of mycoviruses across vegetatively incompatible groups (Wu et al., 2017). SsMYRV4 inhibits expression of heterotrimeric G proteins and *het* or *vic* genes involved in vegetative incompatibility (Wu et al., 2017). Further, the infection of SsMYRV4 reduces cellular reactive oxygen species (ROS), which plays a major role in fungal vegetative incompatibility reactions (Brosché et al., 2014). In *S. sclerotiorum*, infection of SsMYRV4 determines the infection ability of other mycoviruses. Involvement of *vic* genes in vegetative incompatibility was also reported in Chestnut blight fungus, *Cryphonectria parasitica* (Choi et al., 2012; Zhang et al., 2014). Cryphonectria hypovirus 1 (CHV1) infection in *C. parasitica* suppresses expression of the pheromone precursor genes, *Mf1/1, Mf2/1*, and *Mf2/2*, resulting in disturbance in the fungal sexual cycles. The defect in the sexual cycles likely decreases the allelic diverstity of the *vic* gene, thereby promoting the virus transmission among different strains (Zhang et al., 1998).

## Infection by a Mycovirus With an RNA Silencing Suppressor

Many fungi use the adaptive immune system of RNA silencing to suppress viruses (Hammond et al., 2008; Yaegashi et al., 2016). Studies showed that mycoviruses like CHV1 and Rosellinia necatrix mycoreovirus 3 infecting *Cryphonectria parasitica* and *Rosellinia necatrix*, respectively, encode RNA silencing suppressor (RSS) proteins that show similarity to plant RSS proteins (Segers et al., 2006; Yaegashi et al., 2013b). The RSS of one mycovirus may help another mycovirus that gets suppressed by the host RNA silencing, to facilitate coinfection (Chiba and Suzuki, 2015). For example, Rosellinia necatrix victorivirus 1 (RnVV1) originally isolated from *R. necatrix* can replicate in *C. parasitica* when coinfected with CHV1, but not in the virus-free *C. parasitica* strain (EP155). Infection in an RNA silencing mutant of *C. parasitica*, dicer-like 2 knockout-Δ*dcl-2* has a similar effect (Segers et al., 2006; Chiba et al., 2013). This suggests RnVV1 has very low RNA silencing suppressor activities (Chiba et al., 2013). Similarly, RnVV1 replication is impaired when coinfected with *Mycoreovirus 1* (MyRV1) and CHV1 mutant-Δ*p69* in *C. parasitica*. MyRV1 induces silencing genes dicer-like 2 (*dcl2*) and argonaute-like 2 (*agl2*), and CHV1 mutant (Δ*p69*) is also impaired in RSS activity (Chiba and Suzuki, 2015). These examples show that a mycovirus can mediate coinfection of another mycovirus through RNA silencing pathways.

## Nutritional/Chemical Determinants

The roles of chemical compounds that affect the programmed cell death pathways involved in mycelial incompatibility were studied to look at their facilitation of heterologous transmission of mycoviruses. One successful case was the use of a zinc compound *in vitro* that accelerates the transmission of mycoviruses among vegetatively incompatible strains of *R. necatrix* (Ikeda et al., 2013). *Rosellinia necatrix* shows very strong incompatibility with different strains without the formation of anastomosis (Inoue et al., 2011a). Zinc chloride concentrations in the media ranging from 0.5 to 1.5 mM facilitated heterologous transmission of Rosellinia necatrix megabirnavirus 1 and other partitiviruses similar to Rosellinia necatrix partitivirus 3 (Inoue et al., 2011a). How zinc chloride induces anastomosis formation in *R. necatrix* is unknown however, it is inferred that zinc chloride reduces the effects of the hyphal secretion that suppress anastomosis (Inoue et al., 2011a). In the basidiomycete fungus, *Helicobasidium mompa* hyphal incompatibility is inhibited with the supplementation of active charcoal in the media (Inoue et al., 2011b). It is likely that in nature nutrient availability influences the coinfection potential of mycoviruses among fugal hosts through the heterologous anastomosis.

## Conclusions

The determinants of mycovirus coinfections involve multiple factors. One of the constraints that many mycoviruses have to overcome for infection is non-self-recognition. RNA silencing is another major hurdle that influences the success of infection. The roles of nutrients or chemicals or other components of the environment seems to have potential influence in determining infection, but it is a poorly studied field. The biology of coinfection is complex like any other biological phenomena and many factors remained to be explored. On the other hand, it is an important field particularly in manipulating virulence of pathogenic fungi in which mycoviruses are known to play crucial roles. There are attempts underway to alter the non-self-recognition factors in vegetative incompatible populations for successful transfer of mycovirus related to hypovirulence (Zhang et al., 2016). Similar efforts to modify RNA silencing suppressors may prove more difficult. The idea is to produce desired coinfection interactions to manipulate the fungal host's virulence. The coinfection biology of mycoviruses has an important scope not only academically but also in many practical applications associated with hypovirulence, and deserves further attention.

## Author Contributions

VT wrote the first draft. MR edited and wrote the final draft.

### Conflict of Interest Statement

The authors declare that the research was conducted in the absence of any commercial or financial relationships that could be construed as a potential conflict of interest.
